# Site-selective reactions mediated by molecular containers

**DOI:** 10.3762/bjoc.18.35

**Published:** 2022-03-14

**Authors:** Rui Wang, Yang Yu

**Affiliations:** 1 Center for Supramolecular Chemistry & Catalysis and Department of Chemistry, College of Science, Shanghai University, 99 Shang-Da Road, Shanghai 200444, China

**Keywords:** confinement, microenvironment, molecular containers, noncovalent protective group, site-selectivity

## Abstract

In this review, we summarize various site-selective reactions mediated by molecular containers. The emphasis is on those reactions that give different product distributions on the potential reactive sites inside the containers than they do outside, free in solution. Specific cases include site-selective cycloaddition and addition of arenes, reduction of epoxides, α,β-unsaturated aldehydes, azides, halides and alkenes, oxidation of remote C–H bonds and alkenes, and substitution reactions involving ring-opening cyclization of epoxides, nucleophilic substitution of allylic chlorides, and hydrolysis reactions. The product selectivity is interpreted as the consequence of the space shape and environment inside the container. The containers include supramolecular structures self-assembled through metal/ligand interactions or hydrogen bonding and open-ended covalent structures such as cyclodextrins and cavitands. Challenges and prospects for the future are also provided.

## Introduction

To run reactions with discriminate control over product selectivity represents one of the huge challenges in organic synthetic chemistry [1], among which, site-selectivity is always crucial to a reaction when there is more than one potential reactive site in a certain substrate, because poor site-selectivity would result in complicated and sometimes even unachievable separation and purification procedures. Hence, in order to drive reactions economically and efficiently, organic chemists have made great efforts to increase site-selectivity, with the best result of site-specificity [2–4]. However, it is rather difficult to do so, differentiating one certain reactive site from the similar others, because the difference between their transition-state free energies, that would modulate isomeric product ratio, is always small. The selectivity of a reaction depends on its mechanism, and the inherent feature of the substrate should be enhanced or overturned to obtain one certain isomer, with the consideration of electronic, steric, and stereoelectronic factors [5–6]. As a representative strategy, directing groups are introduced to the substrates covalently to achieve site-selective C–H bond activation, which prospered greatly in the past decades [7–9]. Template regulation is also introduced to locate reactive centers in a noncovalent way through hydrogen bonding [10–12]. Even though chemists have developed different kinds of methods to achieve site-selectivity of various reactions, new methodologies that blend with other research fields are still needed.

After decades of rapid development, supramolecular chemistry has won two times for Nobel Prizes and already became one of the most important fields in modern chemistry [13–15]. It is based on a wide range of noncovalent interactions between molecules [16–18] and has been applied to a variety of research areas including molecular recognition, molecular devices, nanochemistry, catalysis, etc. [13–18]. By mimicking the recognition and catalysis behavior of enzymes with designed and synthesized molecular containers such as cyclodextrins, cucurbiturils, calixarenes, and resorcinarenes, chemists try to tackle problems of traditional synthetic chemistry, including increase in reactivity, induction of selectivity, and even emergence of new reaction pathways [19–24]. To simulate the aqueous environment of enzyme-catalyzed physiological transformations, researchers seek to design and synthesize supramolecular hosts in a water-soluble way. The ionic and polyol forms of them would provide good water solubility, and, on the other hand, these kinds of water-soluble moieties could also be introduced into the structures of other hosts to help them gain some extent of water solubility. In aqueous solution, the molecular containers provide hydrophobic pockets capable of binding a wide range of organic compounds. Within the molecular container, guest molecules can be encapsulated with a certain orientation and conformation through various noncovalent interactions. In this mode, the molecular container can act as reaction template and give rise to selective products. For example, the molecular container can be used as anchoring template, which fix the substrate with a certain stable conformation, exposing one specific reactive site to the catalyst and producing site-selective product [25–26]. Moreover, molecular containers have more and more been applied to modulate site-selectivity of different types of reactions and this research field has drawn much attention in the past years [27–30]. It is believed that there is still more to explore and develop in this area, so we summarize representative research works about molecular-container-confined organic reactions with site-selectivity, that is, selective reactions that take place at one specific potential reactive site out of the similar others, in this review. In the following part, the literature reports will be mainly divided according to the reaction types, namely cycloaddition/addition, reduction, oxidation, and substitution.

## Review

### Cycloaddition/addition

**Figure 1:**
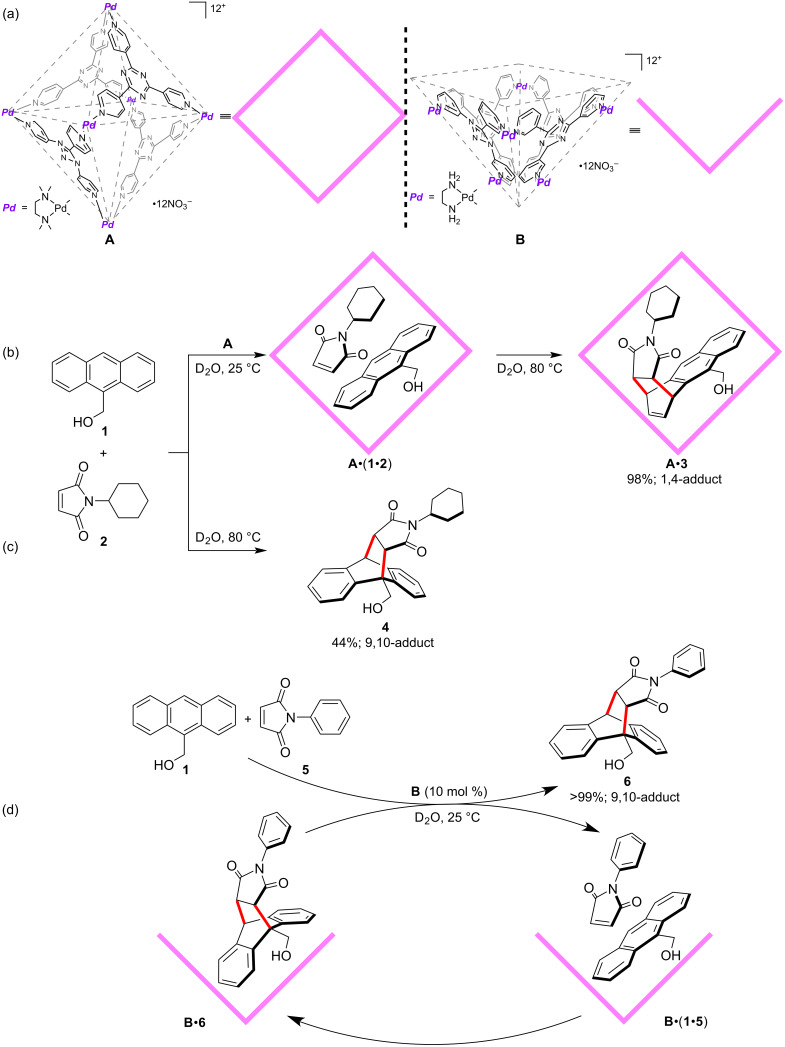
Site-selective Diels–Alder reaction of anthracene and phthalimide mediated by aqueous organopalladium-coordinated hosts **A** and **B**.

Cycloaddition reactions have long been applied to molecular container-mediated enzyme-mimicking transformations [27,31–33], and the Fujita group has done pioneering research works in this direction [27,34]. In 2006, the authors reported unique Diels–Alder reactions of anthracene and phthalimide guests with unusual and controllable site-selectivity mediated by organopalladium-coordinated hosts in water (Figure 1) [34]. The water-solubility of the coordinated host traced from its ionic form, and the aqueous reaction conformed with the concept of green chemistry. In previous reports of supramolecular host-mediated Diels–Alder reactions of anthracenes, 9,10-adducts bridging the center rings of the anthracene frameworks were generally yielded [35–37], which resulted from the high localization of π-electron density at that sites [38]. Besides, these reactions required near-stoichiometric quantities of hosts because of the product inhibition effect, which arose from the entropic disadvantage of the need for binding two reactant molecules [39–43]. In this particular report, when the octahedral cage host **A** was used, the Diels–Alder reaction of 9-hydroxymethylanthracene (**1**) and *N*-cyclohexylphthalimide (**2**) went smoothly at 80 °C for 5 hours with near quantitative yield (Figure 1b). Only the *syn*-isomer of the 1,4-adduct **3** was detected after the reaction, which was determined by X-ray crystallographic analysis of **A**•**3**. It was also shown that the product was tightly accommodated in the cavity of **A** through π–π stacking interactions between the naphthalene ring of **3** and a triazine ligand of **A** from the X-ray crystallographic analysis. In the control experiment, without host **A**, only 44% yield of the conventional 9,10-adduct **4** was produced without any 1,4-adduct product (Figure 1c). This kind of unusual site-selectivity originated from the fixed orientation of the guest substrates confined to the cage host **A** before the reaction. Force-field calculations showed that the guest substrates **1** and **2** were parallel to each other with the double bond of **2** in close contact with the 1,4-position of **1**. On the other hand, the double bond of **2** hardly interacted with the 9,10-position of **1**, because of the steric effect induced by the cage host **A**. This methodology was also compatible with several other anthracene substrates with different substituents at the 9-position. But when the sterically less demanding *N*-propylphthalimide was used, only the 9,10-adduct was formed, which indicated that the steric bulkiness of the *N*-substituent in the dienophile also affected the 1,4-site-selectivity. It is very intriguing that when a different kind of square-pyramidal bowl host **B** was employed, the site-selectivity turned back to the 1,9-position, and with catalytic turnover (Figure 1d). Only 10 mol % of **B** promoted the Diels–Alder reaction of **1** and *N*-phenylphthalimide (**5**) almost quantitatively affording the 9,10-adduct **6** at room temperature for 5 hours. Control experiments proved the promoting effect of the hydrophobic pocket of **B**. The origin of the catalytic behavior of the bowl host **B** can be explained by to two main aspects. Firstly, the bowl host **B** possesses an open cavity that facilitates rapid binding and dissociation of the guests. Secondly and more importantly, before the reaction, the anthracene moiety stacks onto the planar triazine of **B** through π–π stacking and possible charge-transfer interaction between each other, which stabilizes the complex. However, after the reaction, the framework of the product is bent at the 9,10-position, which undermines the host–guest stacking interaction. The complex of **B** and the product is hence destabilized, resulting in the replacement by incoming reactants. This beautiful pioneering work showed elegant examples of how the designed and synthetic molecular containers could mediate and even control the site-selectivity of organic reactions.

**Figure 2:**
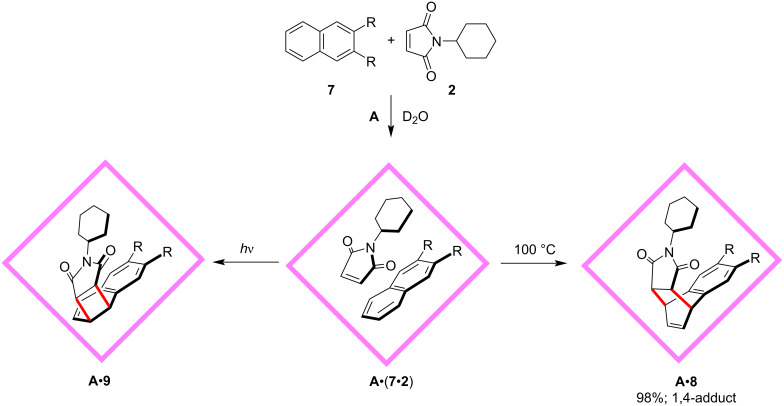
Site-selective Diels–Alder and [2 + 2]-photoaddition reactions between naphthalene and phthalimide mediated by cage host **A**.

Naphthalene is usually hard to undergo Diels–Alder reactions [44–46], even though the quantum-mechanical and thermochemical calculations suggest that the reaction is exothermic, which indicates the entropic cost is significant [47]. In 2010, the same group reported another interesting site-selective Diels–Alder and [2 + 2]-photoaddition reactions between **2** and 2,3-substituted naphthalene **7** mediated by cage host **A** mentioned above (Figure 2) [48]. Given the reduction of the entropic cost resulting from the effective concentration and preorganization of the guest reactants confined to the molecular containers, the authors designed to investigate the Diels–Alder reaction between **2** and naphthalene **7**. As expected, the reactants were encapsulated within the cage host **A** successfully at room temperature and formed a ternary complex **A**•(**7**•**2**), and after being heated at 100 °C for 8 hours, the site-selective product **8** was obtained with moderate yield. No other side reactions were observed, and the moderate yield resulted from the partial sublimation of the reactants. The reaction proceeded site-selectively at the unsubstituted ring of the 2,3-substituted naphthalene, and produced stereoselectively the *syn* isomer **8**, which was determined and confirmed by multiple characterization methods including NMR, mass, and X-ray crystallographic analysis. The reaction did not take place without the cage host **A**. Alkyl substituents at the C2 and C3 position of naphthalene were crucial to this reaction. Control experiments upon the substituent effect indicated that it was the steric, not the electronic factor, that ruled the reactivity and selectivity. The electron-donating alkyl groups should have facilitated reaction at the substituted ring, in contrast, the reaction occurred at the unsubstituted ring. This unusual site-selectivity can be explained by the preorganization of the reactants within the cage host **A**. In the confined space of the cavity, the orientation of the substrates was fixed presumably with the unsubstituted ring of naphthalene subject to the double bond of **2**. This work showed the remarkable function of molecular containers to override natural reactivity and produce unusual site-selective products. Intriguingly, upon irradiation, a site- and stereoselective [2 + 2]-photoaddition between **2** and **7** took place smoothly, giving rise to the *syn* isomer of the 5,6-adduct **9**. In a following work, the authors reported similar site-selective Diels–Alder reactions between **2** and inert aromatics including aceanthrylene and 1*H*-cyclopenta[*l*]phenanthrene [49].

**Figure 3:**
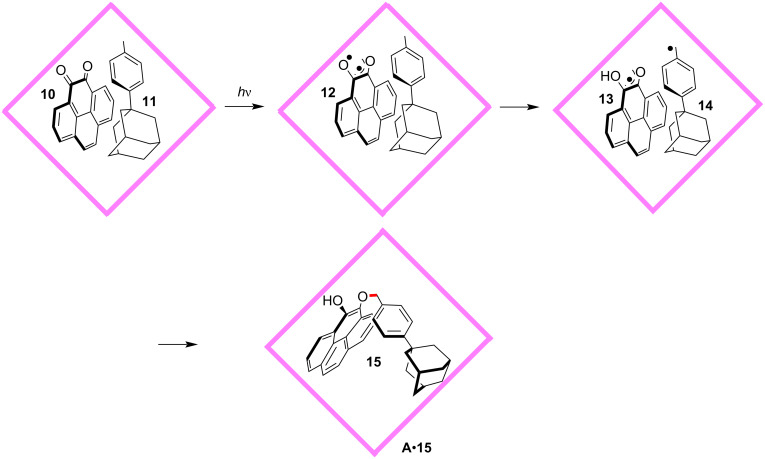
Cage host **A**-mediated selective 1,4-radical addition of *o*-quinone **10**.

Generally, it is difficult to achieve site- and stereoselective control over radical reactions. The radical species are very reactive and a complex mixture of different products will form through various pathways [50–53]. By applying the cage host **A**, the authors realized a highly site-selective radical addition reaction of *o*-quinone **10** and substituted toluene **11**, giving rise to the unusual 1,4-adduct **15** (Figure 3) [54]. Specifically, upon irradiation, biradical species **12** was generated and immediately abstracted a hydrogen atom from the methyl group of **11**. Site-selective radical coupling at the oxygen atom between **13** and **14** produced the 1,4-adduct **15**. The unusual site-selectivity of this reaction was also traced from the restricted geometry and fixed orientation of the guests inside the cage **A**. One of the carbonyl groups of **10** was in close proximity to the methyl group of **11**, which was determined by X-ray crystallography. The possible *C*-coupled 1,2-adduct and other coupling products were not detected. However, in the absence of host **A**, a mixture was formed without the *O*-coupled 1,4-adduct **15**. This indicated that the molecular container **A** favored the *O*-coupling pathway while suppressed others. This work showed the powerful site-selective control ability of molecular containers, which was normally only observed in natural enzymes.

### Reduction

**Figure 4:**
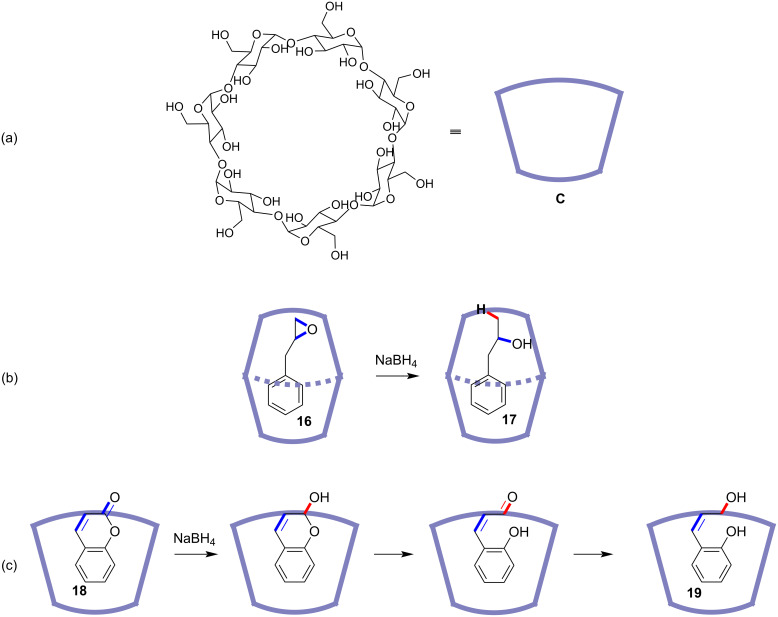
Cyclodextrin-mediated site-selective reductions.

Except for controlled cycloadditions, the site-selective reduction is also difficile to achieve. It mainly depends on the oxidative difference between the potential reactive sites and the careful picking of reductive reagents. Once the oxidative properties of these sites are similar to each other, it is rather hard to just reduce only one site in the presence of the others. Protecting groups are widely used to prevent reaction of one or more functional groups and let others to react [55–57]. Generally, protecting groups are covalently connected to the targeted groups, which requires prefunctionalization and deprotection synthetic procedures. Based on the logical concept of protecting groups, noncovalent interactions can be considered, because they can be built up in situ and are weak enough to let the substrate dissociate from the “protecting template” easily, omitting the complicated prefunctionalization and deprotection processes. Moreover, functional groups that are not suitable for being functionalized with protecting groups can also be incorporated into the noncovalent protective systems. Actually, the molecular container has been applied to work as a noncovalent protective module. In this mode, the molecular container selectively binds with and shields a certain part of the guest molecule and leaves the remaining part exposed to the reaction medium. This methodology was firstly applied to the site-selective reduction reaction mediated by a cyclodextrin host. In 1991, the Takahashi group reported the cyclodextrin-mediated site-selective ring-opening reductive reaction of epoxide **16** by sodium borohydride in aqueous solution (Figure 4b) [58]. The sugar-based hosts show good water solubility and can be used for driving organic reactions in water. In this case, the cyclodextrin host and the epoxide guest formed a 2:1 complex, and the internal reactive site of the epoxide was protected by the cyclodextrin host. Therefore, only the terminal site was attacked by the incoming hydride leading to epoxide-ring opening and formation of 1-phenyl-2-propanol (**17**). Utilizing the similar molecular container as the noncovalent protective group, the Pitchumani and Srinivasan group also reported that the reduction of coumarin (**18**) by sodium borohydride could be site-selectively induced in the presence of β-cyclodextrin **C** (Figure 4c) [59]. The reduction site-selectively occurred at the carbonyl not the alkenyl site, producing the final 1,2-reduction product *cis*-*O*-hydroxycinnamyl alcohol **19**. As a comparison, in the absence of the β-cyclodextrin host, both the 1,2- and 1,4-reduction products were observed. X-ray crystallography determined the host–guest complex of the coumarin and β-cyclodextrin, which could be regarded as a protective group by shielding the internal alkenyl site.

**Figure 5:**
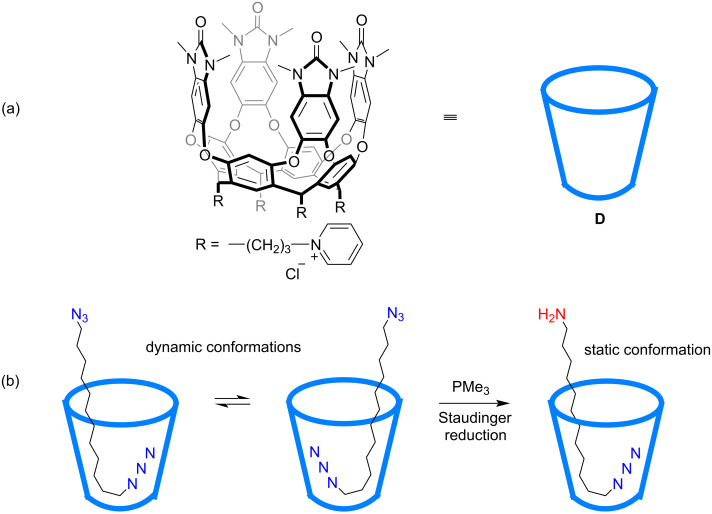
Selective reduction of an α,ω-diazide compound mediated by water-soluble cavitand **D**.

In 2016, the Rebek group achieved the site-selective reduction of an α,ω-diazide compound by trimethylphosphine (PMe_3_) in aqueous solution with a cavitand host as the protecting group for one of the azide sites (Figure 5) [60]. The host in here was a water-soluble deep cavitand **D** with methylated urea groups on the rim, which had already been used to mediate other organic reactions [61]. The feet of the host were transformed to pyridinium cationic moieties to make it soluble in water, and in other examples, similar cavitand hosts were also modified with imidazolium cationic or carboxylic anionic feet [29]. Before the reaction, NMR analysis of the host–guest complex indicated that the bound guest was in yo-yo motions time-averaged between unsymmetrical J-shaped conformations and symmetrical U-shaped ones. Treatment of the complex solution with three equivalents of PMe_3_ resulted in the reduction of one of the azide groups. At this stage, the monoamine guest showed a fixed unsymmetrical J-shaped conformation with the amine end exposed and the azide end deeply protected inside the cavitand. The addition of another 3 equivalents of PMe_3_ to the post-reaction mixture after 24 hours still did not induce further reduction of the residual azide group. However, control experiments gave just the diamine products. This work opened the protective ability of water-soluble cavitands and inspired many other following examples of various site-selective mono-transformations.

**Figure 6:**
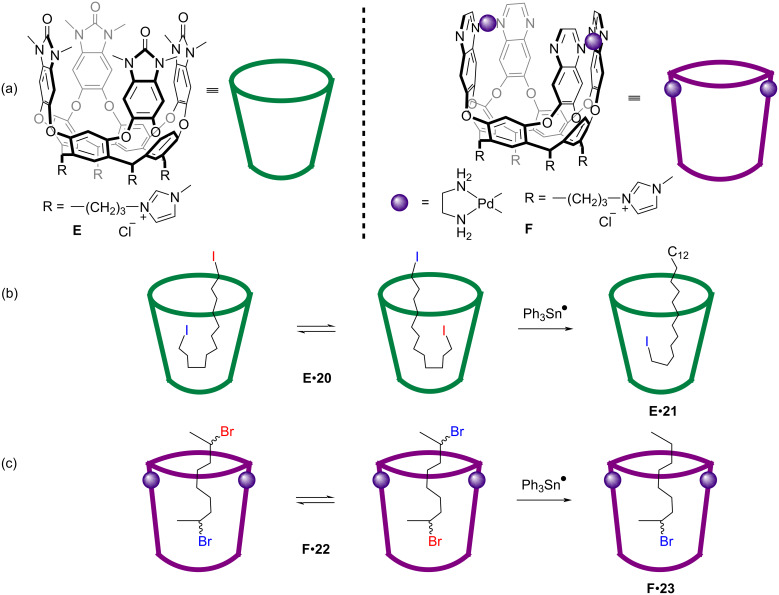
Selective radical reduction of α,ω-dihalides mediated by water-soluble cavitands **E** and **F**.

Another site-selective radical monoreduction of dihalides mediated by water-soluble cavitand hosts **E** and **F** was reported later (Figure 6) [62]. Ph_3_SnH was used as radical initiator and reduction reagent, and alkyl dihalide **20** could be site-selectively monoreduced to the corresponding alkyl halide **21** as major product, together with minor alkane product that arose from the reaction outside the cavitand (Figure 6b). Experiments also indicated that the binding of the guests with the hosts must show high affinities (*K*_A_ > 1.2 × 10^3^ M^−1^) to make sure the reactions occur under confinement in the host. When using a rigidified host **F**, the secondary alkyl dibromide **22** was transformed to the monoreduced product **23** with high site-selectivity, which benefited from the high *K*_A_ value (≈1.5 × 10^5^ M^−1^) (Figure 6c). This work represented the first example of supramolecular containers applied for a radical reaction involving external radical initiators with dynamic hosts. Later, this group reported another highly site-selective radical monoreduction of dihalides by trialkylsilanes (R_3_SiH) using the similar strategy [63].

**Figure 7:**
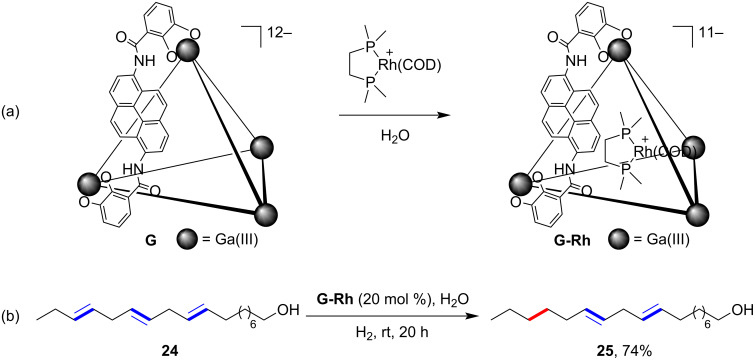
Site-selective hydrogenation of polyenols mediated by supramolecular encapsulated rhodium catalyst.

A very intriguing site-selective catalytic hydrogenation reaction mediated by a supramolecular catalyst was reported by Raymond, Bergman and Toste in 2019 (Figure 7) [64]. In this example, the supramolecular catalyst was prepared in situ by mixing a rhodium complex with the Ga_4_L_6_^12−^ cage host **G**, which had a relatively larger size with pyrene-walled ligands (Figure 7a). The normally used analogous smaller-sized host was assembled with naphthalene-walled ligands, which had been used widely in mediating various reactions, including dehydration reaction [65], aza-Darzens reaction [66], and reductive amination [67], etc. [28]. The anionic cage host demonstrated a relatively high affinity towards cationic guests through cation–π interactions, which was crucial for the catalysis of many of the organic reactions. And similarly, the ionic form of the host made it water-soluble and reactions could be conducted in water. In this particular example, the polyenol substrate **24**, derived from linolenic acid, was monohydrogenated at the terminal, sterically accessible site inside the supramolecular supported catalyst to **25** with 74% yield at room temperature for 20 hours (Figure 7b). A control experiment showed that this kind of site-selectivity could not be achieved with just the rhodium catalyst, by which in contrast, the fully hydrogenated product was obtained. Other series of intermolecular comparative experiments also showed the selectivity of the hydrogenation for the sterically accessible alkene over other sites and even in the presence of inherently more reactive alkynes and allylic alcohols. Both the microenvironment of the supramolecular catalyst and the steric profile of the substrate were responsible for the site-selectivity of hydrogenation. This beautiful work of a supramolecular-mediated catalytic site-selective reaction exhibited the powerful role of molecular containers to achieve precise transformation of complex molecules.

### Oxidation

**Figure 8:**
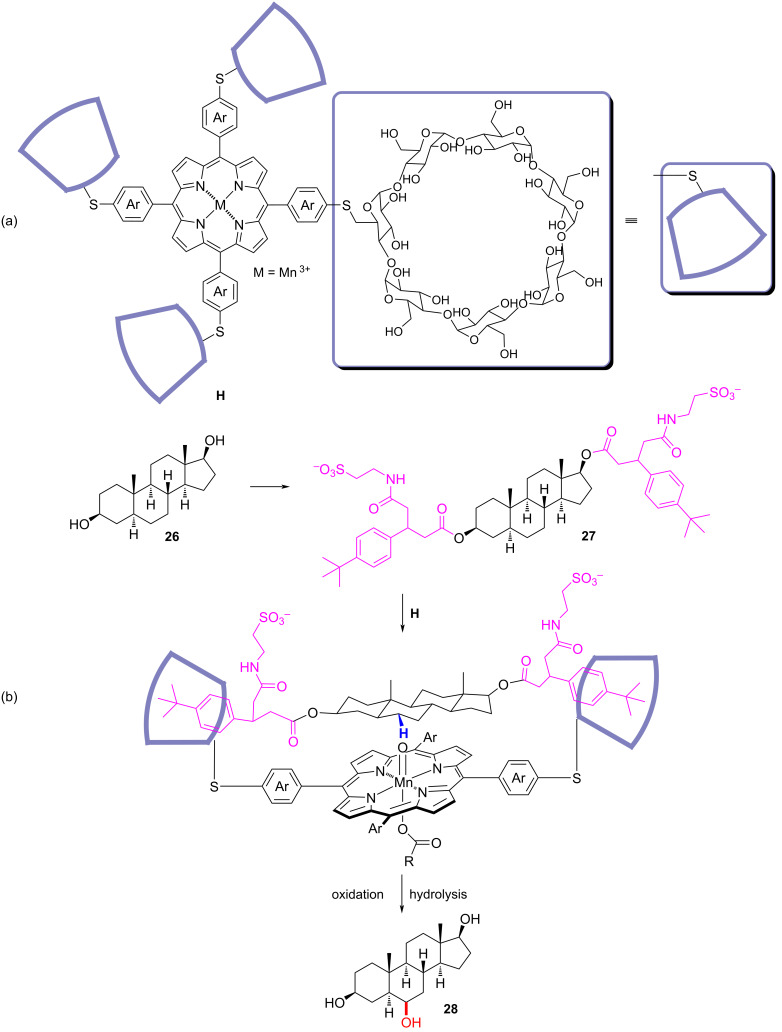
Site-selective oxidation of steroids using cyclodextrin as the anchoring template.

C–H bonds are ubiquitously distributed in nearly all of the organic compounds, which makes them predominant candidates for the modification of complex molecules. Without pre-activation, direct functionalization of C–H bonds brings reaction economy and effectiveness. However, owing to the rifeness of the reactivity-similar C–H bonds, it is hard to achieve discriminate control over the product site-selectivity. In nature, the site-selective oxidation of C–H bonds is facilitated by enzymes with the donor molecules orienting precisely fixed towards the active site of the enzyme through multiple noncovalent interactions [68–69]. Inspired by the magical ability of the enzyme’s receptor site to act on the substrate with fixed orientation, the Breslow group has done a lot of leading works [25–26] utilizing cyclodextrin as the anchoring template. For example, cyclodextrin would fix the steroid substrate with a certain set of orientation, which exposes one certain C–H bond to the metalloporphyrin catalytic moiety and produces site-selective oxidized product. As shown in Figure 8, in this methodology, the steroid substrate **26** was first modified at the hydroxy groups through esterification with a designed acid moiety possessing a *p*-*tert*-butylphenyl group and transformed to the corresponding model substrate **27**. The catalyst **H** used here was a manganese(III)-bounded porphyrin module carrying four β-cyclodextrin units at the end. Once the two parts were mixed in the reaction system, the two *p*-*tert*-butylphenyl groups of the substrate **27** were recognized by the β-cyclodextrin and anchored through the host–guest binding. At this stage, the steroid core exposed the 6-position C–H bond to the metalloporphyrin unit catalytic center and gave rise to the site-selective 6-hydroxy derivative, which was then hydrolyzed to the final site-selective product **28**. The series of work showed that the powerful molecular container could be used as the anchoring group in the template catalysis. Subsequent reports by other groups illustrated that apart from molecular containers, other designed moieties could also be used to anchor the substrate through hydrogen-bonding interactions [10–12].

**Figure 9:**
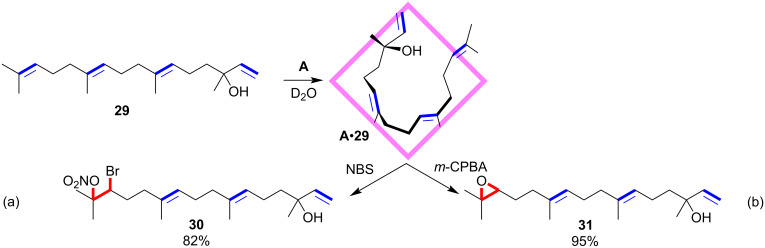
Site-selective oxidations of linear diterpenoids with the help of cage host **A**.

In 2019, the Fujita group reported the site-selective oxidations of linear diterpenoids with the help of cage host **A** (Figure 9) [70]. The linear diterpenoid substrates have four C–C double bonds with a trisubstituted terminal one. Functionalization of these structures would result in mixtures of products derived from each potential alkene group without site-selectivity. The cage host **A** was proved to recognize organic molecules in water and pre-organize them with certain confined conformations. In this case, the linear diterpenoid substrate **29** possessed a folded U-shaped conformation within the cavity, with the terminal trisubstituted olefin exposed to the solution while the other three internal ones were protected by the cage host. The structure of the host–guest complex was determined by NMR and X-ray crystallographic analysis. Accordingly, the terminal trisubstituted olefin moiety was site-selectively transformed to the corresponding nitratobrominated compound **30** (Figure 9a) or epoxide **31** (Figure 9b) by NBS or *m*-CPBA, respectively. The uncommon nitratobrominated product was speculated to form through the attack of NO_3_^−^ ions, whose concentration was high around the cationic cage host, on the bromonium intermediate. Control experiments indicated the crucial noncovalent protective role of the molecular container to induce site-selectivity.

**Figure 10:**
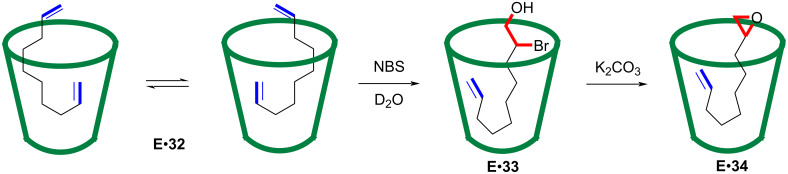
Site-selective monoepoxidation of α,ω-dienes mediated by the water-soluble cavitand host **E**.

Contemporaneously, the Rebek group illustrated another interesting site-selective monoepoxidation of α,ω-dienes mediated by the water-soluble cavitand host **E** (Figure 10) [71]. The α,ω-dienes were determined to adopt a yo-yo motion between two J-shaped conformations or the rapid tumbling of a coiled conformation. For longer guests, it is more like the former type, and for shorter guests, the latter is more likely. In both cases, the two terminal olefins rapidly exchanged positions between the top and the bottom of the cavitand. Treatment of the complex solution of **E**•**32** with NBS produced the monobromohydrin intermediate **33**, which converted to the final site-selective monoepoxide product **34** with the addition of base solution. Control experiments demonstrated the crucial role of the cavitand host to achieve site-selectivity. In this mediation mode, after the monofunctionalization, the more hydrophilic alcohol terminal was exposed to the aqueous solution and the more hydrophobic olefin terminal was buried deep in the cavitand hence protected from the further functionalization. This methodology could be expanded generally to the systems of converting symmetrical hydrophobic guests to unsymmetrical, amphiphilic ones.

### Substitution

**Figure 11:**
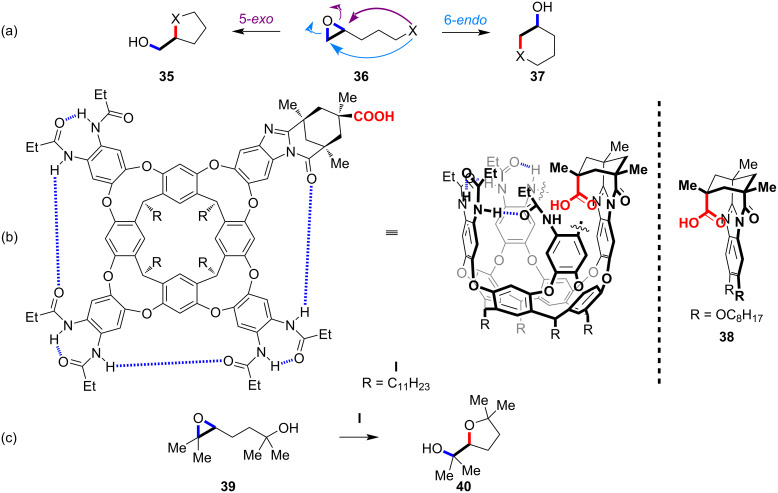
Site-selective ring-opening reaction of epoxides mediated by cavitand **I** with an inwardly directed carboxylic acid module.

In 2008, the Rebek group reported a very intriguing site-selective ring-opening reaction of epoxides mediated by cavitand host **I**, which possessed an inwardly directed carboxylic acid module (Figure 11) [72]. The ring-opening of substituted epoxides results in regioisomeric products, for example, the cyclization ring-opening of epoxide **35** could produce 5- or 6-membered products **36** and **37** via a 5-*exo* or 6-*endo* mode, respectively (Figure 11a). The cavitand host **I** used here was a deep open-ended receptor functionalized with a Kemp’s triacid derivative, which presented the recognized guest molecule with an inwardly directed carboxylic acid group. The hydrogen bonds provided by a cyclic array of secondary amides around the rim stabilized the vase-like conformation of the complex (Figure 11b). Adding the epoxyalcohol **39** to the solution of **I** formed 5-membered ring product **40** exclusively (Figure 11c). However, a control experiment using the model acid **38** afforded a mixture of 5- and 6-membered products. The introduced acid module facilitated the ring-opening reaction, and the CH–π interactions between the aromatic walls of the host and the alkyl backbone of the substrate induced the coiling conformation, giving rise to a compressed 5-membered-ring transition state. Later, the authors offered a detailed discussion regarding the mechanism of this reaction [73]. This example of using a synthetic receptor to achieve site-selective reaction fully showed how a molecular container could mimic the catalysis behavior of the active sites of enzymes.

**Figure 12:**
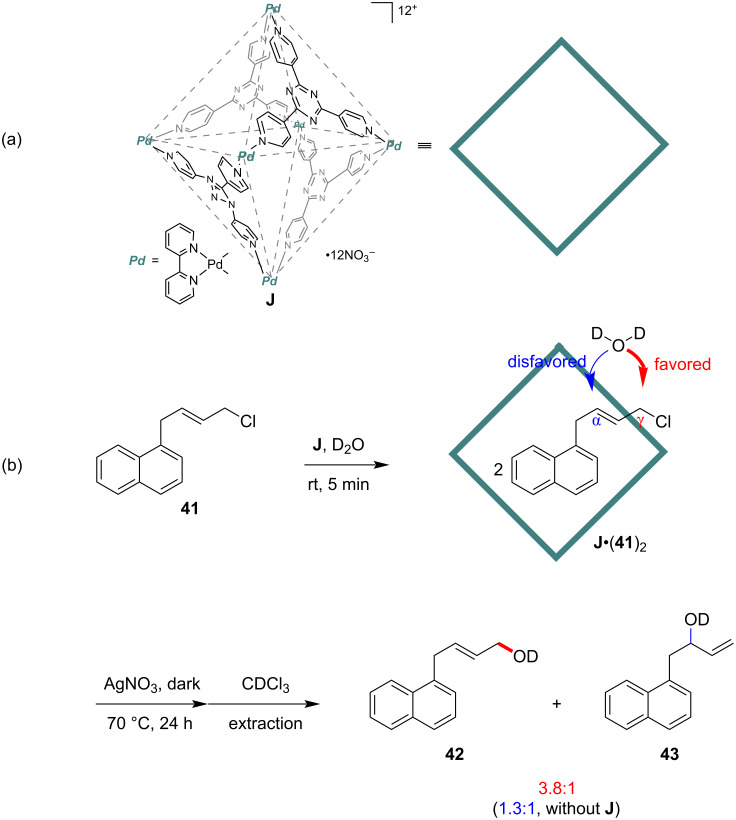
Site-selective nucleophilic substitution reaction of allylic chlorides mediated by cage host **J**.

In 2012, the Fujita group reported a site-selective nucleophilic substitution reaction of allylic chlorides mediated by cage host **J** (Figure 12) [74]. Usually, this reaction occurs both at the α and γ positions of the allylic chloride, and factors like steric and electronic effects of the nucleophile and substrate and the polarity of the solvent would influence the product ratio [75]. Here, as illustrated above, the authors introduced the cage host **J** as the noncovalent protecting group of the internal reactive sites, which directed the incoming nucleophile (D_2_O) to attack at the terminal site and presented terminal-induced product ratio (from 1.3:1 to 3.8:1) (Figure 12b). In this supramolecular system, two guest molecules of **41** were encapsulated per cage to form the **J**•(**41**)_2_ complex. Substrate screening showed the induction effect of the reaction to terminal site-selectivity compared to the reaction in the absence of cage **J**. Even though the induced terminal site-selectivity in this work was not significant, it set an early example of how the molecular container could be beautifully used as a noncovalent protective group for substitution.

**Figure 13:**
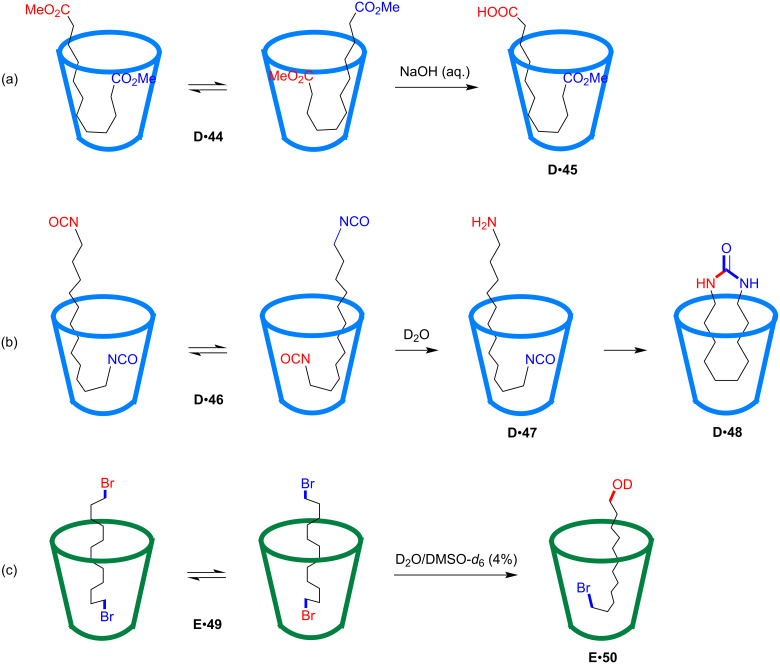
Site-selective monohydrolysis of α,ω-difunctional compounds using deep water-soluble cavitands.

The Rebek group later reported a series of site-selective monohydrolyses of α,ω-difunctional compounds using deep water-soluble cavitands **D** and **E** (Figure 13) [76–78]. As shown in Figure 13a [76], α,ω-diester **44** showed rapidly exchanging and folded J-shape conformations, which exposed each ester group in turn to the aqueous solution. Addition of base would result in the hydrolysis of one ester group to the corresponding carboxylic acid. Product distributions indicated a two- to four-fold relative decrease in the hydrolysis rate constant of the second ester caused by the confined space in the cavitand, which enhanced the selectivity of the monoester product **45**. Similarly, the monohydrolysis of α,ω-diisocyanate **46** was achieved using the water-soluble cavitand **D** (Figure 13b) [77]. The residual isocyanate group was buried deep in the cavity of **D** and protected from further hydrolysis. The monoamine product **47** further underwent intramolecular cyclization facilitated by the confinement of the cavitand and produced cyclized urea product **48**. In a following work (Figure 13c) [78], the binding dibromide **49** showed rapid tumbling conformation in the cavity of the cavitand host **E** on the NMR timescale. DMSO was introduced as a co-solvent to promote the S_N_2-type reaction, and the dibromide **49** was smoothly hydrolyzed site-selectively to the monohydroxy product **50**, which was fixed in an unsymmetrical manner in the cavitand. The bromide terminal was hence protected deep within the cavity of the cavitand host **E** from further hydrolysis, despite the addition of 10 equivalents DMSO after 2 days or even after one month. The experiment without the cavitand host produced a mixture of different products, and the dibromide was fully converted to the dihydroxy product after prolonging the reaction time or increasing DMSO concentration, without the detection of any monohydrolyzed product. This work further demonstrated the striking ability of the water-soluble cavitand to mediate site-selective reactions.

## Conclusion

To summarize, we have reviewed various site-selective reactions mediated by molecular containers, which have drawn much attention in the past years and shown broad prospects in the future. The supramolecular cavity and its constrained microenvironment resemble the active site of natural enzymes, where the guest substrate is encapsulated and positioned with a specific fixed orientation and conformation through various noncovalent interactions, giving rise to discriminate control over product selectivity. In some cases, the molecular container is considered as a noncovalent protective group that prevents potential reactive sites from reacting with external reagents; in other cases, the molecular container acts as the anchoring template and fixes the substrate with a certain conformation, exposing one reactive site to the catalyst center and producing site-selective products. Even though molecular containers have been proved to be powerful tools in inducing reaction selectivity, there are still some restrictions that should be considered. For example, the substrate scopes of these methodologies are generally limited; sometimes, a near quantitative amount of the supramolecular host is required and the synthesis of macromolecular hosts is sometimes complicated. Future research should focus on those limitations as well as developing diverse catalysis systems that would induce controllable site-selectivity.
